# An In Situ Electrical Impedance Tomography Sensor System for Biomass Estimation of Tap Roots

**DOI:** 10.3390/plants11131713

**Published:** 2022-06-28

**Authors:** Rinku Basak, Khan A. Wahid

**Affiliations:** Department of Electrical and Computer Engineering, University of Saskatchewan, Saskatoon, SK S7N 5A9, Canada; khan.wahid@usask.ca

**Keywords:** electrical impedance tomography, image reconstruction, plant phenotyping, root growth, root biomass

## Abstract

Root biomass is one of the most relevant root parameters for studies of plant response to environmental change. In this work, a dynamic and adjustable electrode array sensor system is designed for developing a cost-effective, high-speed data acquisition system based on electrical impedance tomography (EIT). The developed EIT system is found to be suitable for in situ measurements and capable of monitoring the changes in root growth and development with three-dimensional imaging by measuring impedances in multiple frequencies with the help of an EIT sensor. The designed EIT sensor system is assessed and calibrated by the inhomogeneities in both water and soil media. The impedances are measured for multiple tap roots using an electrical impedance spectroscopy (EIS) tool connected to the sensor at frequencies ranging from 1 kHz to 100 kHz. The changes in conductivity are calculated by obtaining the boundary voltages from the measured impedances for a given stimulation current. A non-invasive imaging method is utilized, and the spectral changes are observed accordingly to evaluate the growth of the roots. A further root analysis helps us estimate the root biomass non-destructively in real-time. The root size (such as, weight, length) is correlated with the measured impedances. A regression analysis is performed using the least square method, and more than 97% correlation is found for the biomass estimation of carrot roots with an RMSE of 4.516. The obtained models are later validated using a new and separate set of carrot root samples and the accuracy of the predicted models is found to be 93% or above. A complete electrode model is utilized, and the reconstruction analysis is performed and optimized by utilizing the impedance imaging technique in difference method. The tomography of the root is reconstructed with finite element method (FEM) modeling considering one-step Gauss–Newton (GN) algorithm which is carried out using an open source software known as electrical impedance and diffuse optical tomography reconstruction software (EIDORS).

## 1. Introduction

EIT is a non-invasive imaging technique by which the changes in conductivity distributions are reconstructed. Electrical conductivity images of any closed domain under test can be reconstructed by knowing the boundary potentials [1,2,3]. The sensor system consists of an electrode array with a single layer for two-dimensional (2D) or multiple layers for three-dimensional (3D) matter. The current density is distributed inside the medium based on the stimulated current through the electrodes. Current excitation and voltage acquisition are repeated continuously and rapidly until all the independent electrode combinations are deployed. The data obtained by measuring all possible impedances are used to reconstruct an image, which provides qualitative and quantitative information of the matter.

A multifrequency EIT (MFEIT) system gives more useful information about biological matters because the electrical voltage appearing across the matter is frequency dependent [4,5,6]. Significant information of the matter can be obtained by injecting currents in multiple frequencies. A good conductivity distribution of an object can be mapped in the domain under test by measuring the electrical impedances through the multiple electrodes at various frequencies. The image of the object can be reconstructed by calculating the boundary potentials for homogeneous and inhomogeneous conditions of the domain. EIT problems can be solved numerically using finite element method (FEM) modeling [3,7]. In forward solve, the boundary potentials are calculated by the injected current and known conductivity in the EIT domain. On the other hand, the unknown conductivity changes are calculated in the inverse solve by knowing the differences in boundary potentials for the given stimulation current in the domain. EIT has high temporal resolution, but poor spatial resolution, so this technique is sensitive to noise [7]. The spatial resolution can be improved by increasing the number of electrodes in the EIT sensor system and by choosing the correct drive pattern upon selection of electrode pairs for current and voltage stimulation [8,9]

A multifrequency impedance imaging technique considers multi-electrode array (eight or more electrodes) in a system. The estimation performance can be potentially improved by considering multispectral impedance imaging using an EIT system [4,5,6]. EIT is a radiation free and cost-effective alternative to other laboratory-based radiative imaging methods such as magnetic resonance imaging (MRI), computed tomography (CT), and positron emission tomography (PET) [10,11,12]. The physiological information of the matter can be obtained from EIT images non-destructively, which can be used for real-time monitoring [9]. Due to its unique advantages, EIT has become useful in applications in the area of biomedical imaging, biotechnology, nanotechnology, and plant physiology [4,5,6,8,9,10,11,12]. Previously, several EIT data acquisition systems were developed for medical applications; 2D imaging was performed and utilized in health monitoring [13], diagnosis of human body diseases [14], and clinical imaging [15]. EIT image reconstruction was studied, and successful evaluation was made considering different algorithms [7,16,17,18,19]. An efficient multi-task structure for multifrequency EIT [7], brain imaging [20], anomaly detection [21,22], and cell imaging [23] using EIT were studied. EIT was also used for crop root systems in agriculture in a limited capacity [10,11,12].

The root system is complex as it provides water and nutrients which are required for photosynthesis to the plant stems and leaves by absorbing them from soil. An investigation into crop root traits, and the growth, development, and biomass of the root is very important in plant phenotyping. The investigation can be made by varying the electrical parameters, such as capacitance, resistance, or impedance. Identifying the size, shape, and structure of the roots is very important in biological study of the plant characteristics, and their electrical parameters were measured [24,25,26,27]. Analyses of root growth [24], recovery of the root body of a plant system in water [25], and the estimation of root biomass [26] were made previously by measuring the electrical capacity. Newill et al. imaged the water distribution in the root zone using the capacitively coupled impedance measurement technique which was acquired at excitation frequencies from 10 Hz to 13 MHz using a Hewlett Packard 4192A impedance analyzer [27]. The measurements were made on laboratory-scale rhizotron containers with a static array of 60 electrodes in a soil media and recovered 2D information only. In addition, the required instrumentation for the experiments was found to be very costly.

In several experiments, the dependency of electrical impedance on plant characteristics was evaluated by varying multiple frequencies and the tomography results were obtained [10,11,12]. Weigand and Kemna utilized a multifrequency EIT in a laboratory for characterizing and monitoring an oilseed root system in a water-filled rhizotron [10]. Imaging and characterization of the oilseed root using spectral EIT considering polarization effects were also carried out using water [11]. The measurements were limited to water media with a maximum operating frequency of 45 kHz. The method only recovered 2D information of the root system in a rhizotron container with a static array of 38 electrodes at the laboratory scale. In addition, the method required expensive instrumentation. On the other hand, Corona-Lopez et al. visualized the developing root system of oilseed rape plants in a compost-filled container using EIT [12]. Some 3D information of the root system in a container with a static array of 32 electrodes was recovered in a limited capacity with a low operating frequency of 5–10 kHz. No information was found about the high spectral reconstructions. During growth and development, the changes in root characteristics can be evaluated by the measured impedances considering frequency difference and time difference EIT [7,22,28]. More information of the roots can be obtained using 3D imaging.

A new non-invasive, cost-effective, and high spectral range 3D EIT system, along with the existing measurement methods, is still a constant requirement in the field of root study. The ability to characterize the root in both water and soil media, to perform non-invasive imaging using 3D at the laboratory and field scale, to monitor growth in dimensions, and to estimate the biomass is still lacking in the EIT system. EIT using a multiple electrode array with the capability of in situ measurements seems to be a promising method to fulfill the scope of further research on plant root tomography. Hence, a new EIT sensor system with dynamic and adjustable electrode array is designed in this work for developing a low-cost, in situ 3D EIT data acquisition system with the target of rapid biomass estimation of tap roots by non-destructive impedance measurements in multiple frequencies.

## 2. Results

### 2.1. Fresh Weight Biomass Estimation

**Figure 1:**
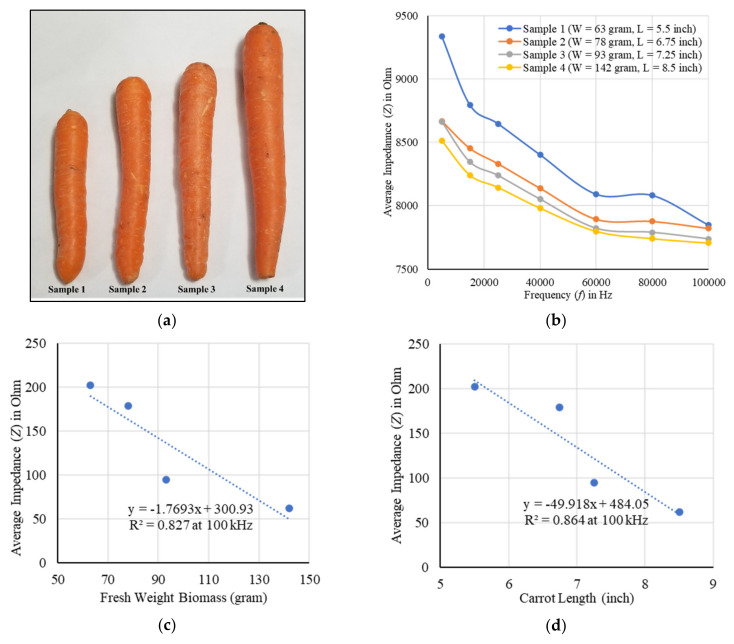
(**a**) Carrot samples, (**b**) the EIS of four different carrot samples in soil, and correlation with (**c**) biomass weights, (**d**) lengths of the samples at 100 kHz. A more than 90% correlation was found between the actual average impedance and the root size (weight and length) of the samples.

The root system of a carrot is tap root and to evaluate the growth of the root in different days of the growing stage four different carrot samples of various fresh biomass weights (W = 63, 78, 93, and 142 g) and lengths (L = 5.5, 6.75, 7.25, and 8.5 inches) were inserted in a given area of soil. The impedances were measured using the designed electrode array sensor system with and without the carrot in soil for 2 Vpp excitation, and the difference was taken to obtain the actual impedance of the carrot. The impedances were measured at different positions of the soil electrodes from top to bottom layers by varying frequency from 5 kHz to 100 kHz. The average impedance for the lower sized carrot (sample 1) in soil was found to be very high, on the other hand, a lower impedance was found for the higher sized carrot (sample 4) in soil as shown in Figure 1. The actual average impedances of the carrots were correlated with the fresh biomass weights and lengths of the carrots at different frequencies. The impedance was decreased with the increase of biomass weight and length of the carrot as shown in Figure 1c and Figure 1d, respectively. A maximum correlation (negative) of more than 90% coefficient was found in both cases at 100 kHz.

**Figure 2:**
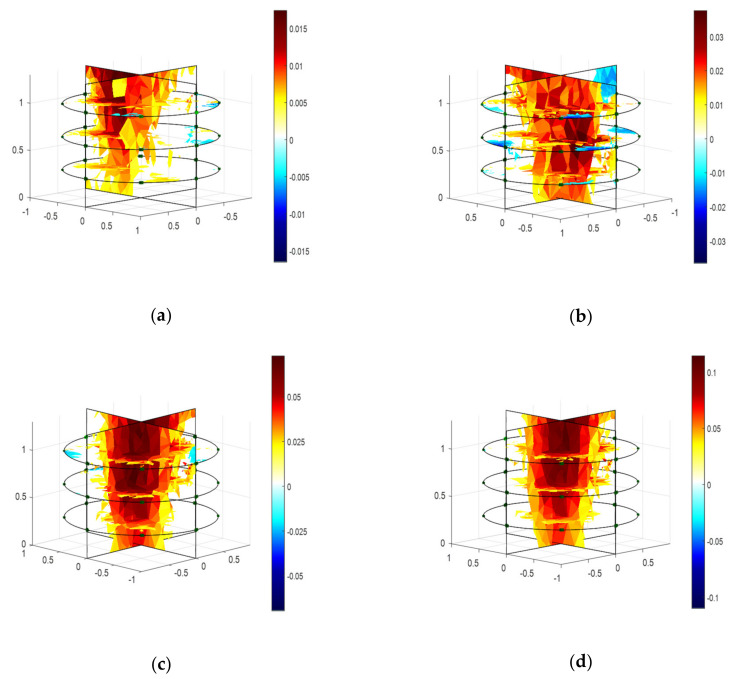
Tomography of four different carrot samples in soil media at 100 kHz, 1 mA, and 2 Vpp excitation considering NOSER (*λ* = 2.17) in the inverse model. (**a**–**d**) The scale represents the changes in conductivity. The conductivity for the lower sized carrot (sample 1) was found to be very low, on the other hand, a highest conductivity was found for the higher sized carrot (sample 4). (**a**) Sample 1 at 100 kHz (W = 63 g, L = 5.5 inch); (**b**) Sample 2 at 100 kHz (W = 78 g, L = 6.75 inch); (**c**) Sample 3 at 100 kHz (W = 93 g, L = 7.25 inch); (**d**) Sample 4 at 100 kHz (W = 142 g, L = 8.5 inch).

To visualize the developing root system and associated changes in soil conductivity, 3D reconstructions of the samples were performed. The impedance tomography of the samples was obtained using the difference method applying one-step GN algorithm as shown in Figure 2. The boundary voltages were obtained from the measured impedances in homogeneous and inhomogeneous conditions of the EIT domain, and those were utilized to obtain the reconstructed images of the samples. The changes in conductivity were calculated from the difference of normalized boundary voltages at 100 kHz and 1 mA using NOSER (*λ* = 2.17). The adjacent tomography results represent the variation of changes in conductivity by varying the sample size (weight and length). The conductivity was increased by increasing the biomass weight and length of the samples. Hence, it is evident that the increases in conductivity with the increase of sample size indicates the growth and development of the root system in soil with time.

In addition, the biomass of the root samples can be classified as very low, low, medium, and high from the obtained maximum conductivity changes of 0.017, 0.038, 0.07, and 0.11, respectively, as shown in Figure 2. The measured average impedance considering sample 1 in the soil media was found to be much higher than the other samples (Figure 1b). Hence, the calculated conductivity changes for sample 1 was found to be much lower than the others. On the other hand, the obtained results show the highest conductivity changes for sample 4. The shape of a root sample can also be represented by the variation in conductivity in top to bottom layers of the tomography. A noisy and deteriorated shape in the reconstructed image was found for sample 1 (Figure 2a), and comparatively less noise with better shape in the reconstructed image was found for sample 4 (Figure 2d). A moderate conductivity and a satisfactory result in shape was found for sample 2 (Figure 2b), and sample 3 (Figure 2c), respectively. Therefore, the EIT sensor is found to be capable of evaluating the growth and development of the plant root by obtaining the changes in conductivity and other associated information from tomography results.

### 2.2. Dry Weight Biomass Estimation

**Figure 3:**
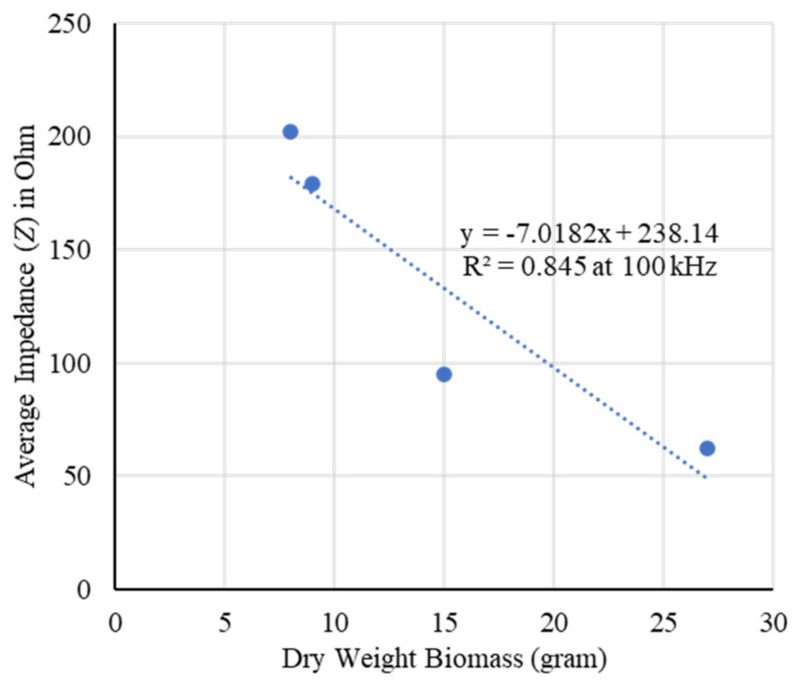
Correlation between dry weight biomass and the actual average impedance of the carrot samples at 100 kHz. An improved correlation with *R*^2^ = 0.845 was found by removing the moisture of the samples.

The carrot samples were then oven dried at 110 °C for six hours and the biomass weights were reduced to 8, 9, 15, and 27 g, respectively, along with the dimensions. By measuring impedances an improved correlation with *R*^2^ = 0.845 at 100 kHz was found for dry weight biomass estimation of the samples as shown in Figure 3. Biomass in terms of fresh weight shows variations in weight due to seasonal moisture differences whereas in terms of dry weight it remains unaffected, and the model is considered more accurate.

### 2.3. Modeling and Estimation of Actual Biomass Weight

**Figure 4:**
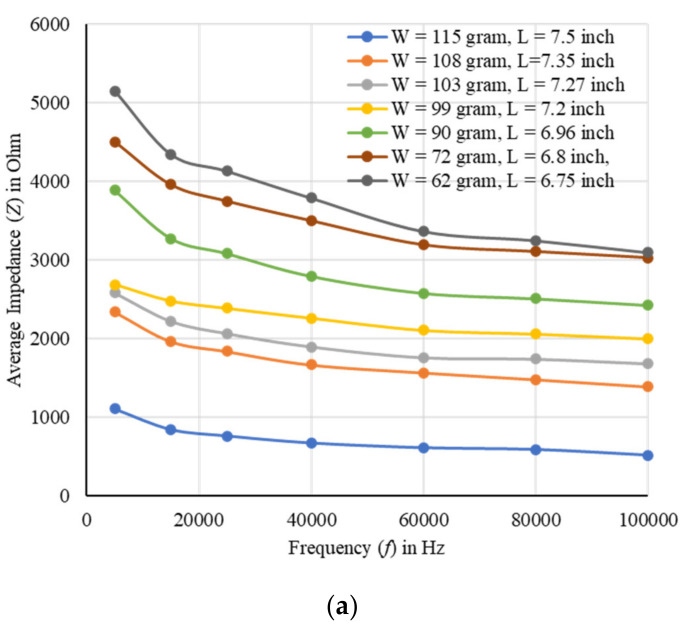

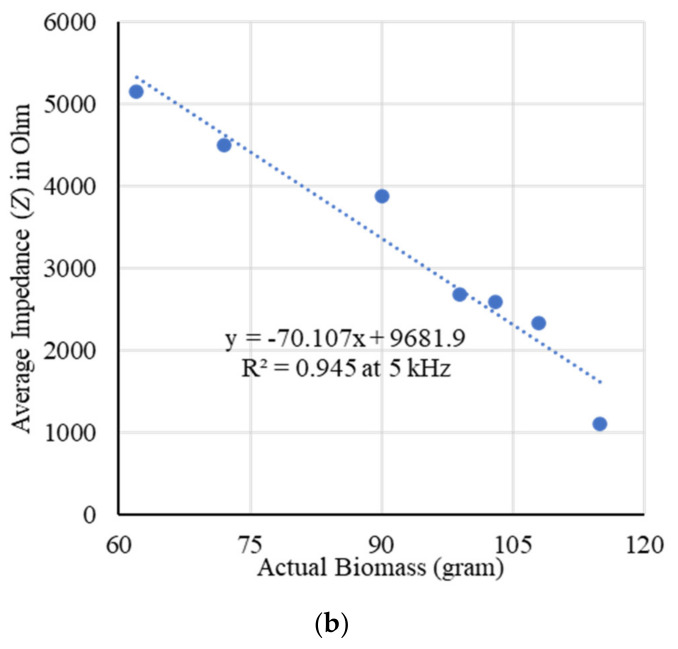
(**a**) The EIS of a carrot in soil for different biomass weights. (**b**) Correlation between actual biomass and average impedances (*Z*) of a carrot, and a maximum correlation of 97.2% (*R*^2^ = 0.945) was obtained at 5 kHz.

In another experiment, the actual biomass weight and the length of a carrot root were taken in different days. The impedances were measured in soil with and without the carrot using three different layers of the designed electrode array. From the difference the carrot impedance (*Z*) was calculated at different frequencies of 5, 15, 25, 40, 60, 80, and 100 kHz, respectively. The fresh weight and length of the carrot were 115 g and 7.5 inches, and after drying at room temperature (20 °C) for 10 days those were reduced to 62 g and 6.75 inches, respectively. The actual average impedance was found to increase with the decrease of biomass weight of a carrot as shown in Figure 4. A good correlation was found at different frequencies, and a maximum correlation of 97.2% (*R*^2^ = 0.945) was obtained at 5 kHz (Figure 4b).

**Figure 5:**
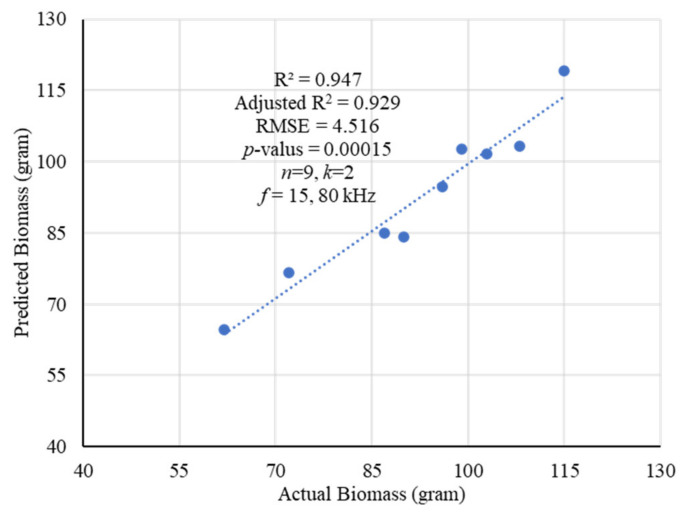
The regression results for predicting the biomass of carrot roots at frequencies of 5 kHz to 100 kHz. More than 97% correlation was obtained with RMSE of 4.516 by selecting the features of 15, and 80 kHz in the model.

The multiple linear regression was employed for predicting the carrot biomass as shown in Figure 5. A strong correlation with *R*^2^ = 0.947 and RMSE of 4.516 was found for *n* = 9 samples, and a model was extracted as shown in Equation (1). Two features (*k*) of 15, and 80 kHz contributed high correlation. The calculated carrot impedances were then normalized (*Z_n_*), and a new model was extracted as shown in Equation (2). Later, layer-wise mean and standard deviation of the normalized impedances were taken to obtain the regression models for predicting the carrot root biomass weight at different frequencies. The models obtained for frequencies of 5 kHz to 100 kHz and those are presented in Equations (3)–(9) considering ridge regression analysis.

(1) $$\hat{Y}=134.398-0.0446Z_{15\mathrm{kHz}}+0.03822Z_{80\mathrm{kHz}}$$

(2) $$\hat{Y}=243.513-2836.9Z_{n15\mathrm{kHz}}+2037.23Z_{n80\mathrm{kHz}}$$

(3) $${\hat{Y}}_{5\mathrm{kHz}}=-96.087-233.21Z_{nl1m}-128.67Z_{nl1SD}+49.9868Z_{nl2m}-129.91Z_{nl2SD}+1450.2Z_{nl3m}-1368.9Z_{nl3SD}$$

(4) $${\hat{Y}}_{15\mathrm{kHz}}=-55.906-169.66Z_{nl1m}-206.4Z_{nl1SD}+51.861Z_{nl2m}-150.46Z_{nl2SD}+1159.67Z_{nl3m}-1298.9Z_{nl3SD}$$

(5) $${\hat{Y}}_{25\mathrm{kHz}}=-33.322-279.12Z_{nl1m}-201.35Z_{nl1SD}+67.6375Z_{nl2m}-159.93Z_{nl2SD}+1112.1Z_{nl3m}-1301.1Z_{nl3SD}$$

(6) $${\hat{Y}}_{40\mathrm{kHz}}=126.109+26.5157Z_{nl1m}-11.87Z_{nl1SD}+73.5124Z_{nl2m}-135.26Z_{nl2SD}-133.24Z_{nl3m}-780.57Z_{nl3SD}$$

(7) $${\hat{Y}}_{60\mathrm{kHz}}=98.7354-126.86Z_{nl1m}-49.358Z_{nl1SD}+38.4318Z_{nl2m}-120.8Z_{nl2SD}+209.306Z_{nl3m}-966.06Z_{nl3SD}$$

(8) $${\hat{Y}}_{80\mathrm{kHz}}=120.781+17.849Z_{nl1m}-43.993Z_{nl1SD}+3.395Z_{nl2m}-98.86Z_{nl2SD}-18.32Z_{nl3m}-838.4Z_{nl3SD}$$

(9) $${\hat{Y}}_{100\mathrm{kHz}}=110.897+126.187Z_{nl1m}-89.055Z_{nl1SD}-3.9723Z_{nl2m}-94.445Z_{nl2SD}-61.193Z_{nl3m}-885.88Z_{nl3SD}$$

**Figure 6:**
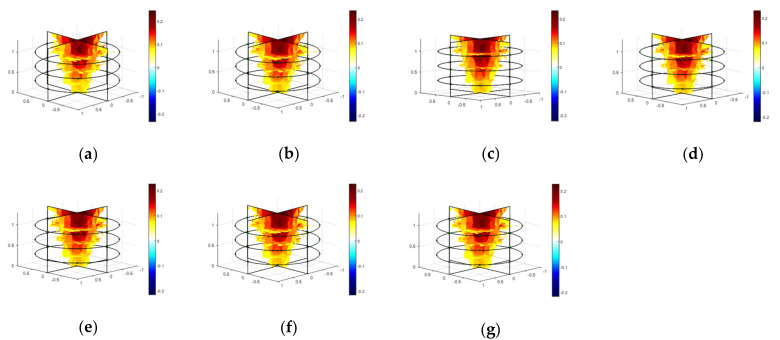
Tomography of the reconstructed carrot of 6.75 inches in length using 3D EIT measurements at 5 kHz to 100 kHz, 1 mA, and 2 Vpp excitation considering NOSER (*λ* = 2.17) in the inverse model. (**a**–**g**) The scale represents the changes in conductivity. (**a**) 5 kHz; (**b**) 15 kHz; (**c**) 25 kHz; (**d**) 40 kHz; (**e**) 60 kHz; (**f**) 80 kHz; (**g**) 100 kHz.

All the models were trained and validated using F-test considering *p*-value less than 5%. The average RMSE was found to be less than 4 with high correlation of *R*^2^ = 0.98 or above and adjusted *R*^2^ = 0.94 or above as shown in Table 1. The tomography results of the carrot of 6.75 inches in length and with a weight of 62 g in a given area of soil for different frequencies of 5 kHz to 100 kHz, 1 mA, and 2 Vpp excitation are presented in Figure 6. A good image reconstruction at different frequencies was made by the impedance measurements using the developed EIT data acquisition system. More useful information about the root sample can be obtained by the measured impedances in multiple frequencies. Although a close approximation result in conductivity was found, the variations can be evaluated by the predicted models in different frequencies. In addition, the layer-wise changes in conductivity along with any variation in dimensions of the sample can also be evaluated by making frequency difference.

The obtained models were then validated as shown in Table 2. Seven new carrot samples of different biomass weights, W = 142, 115, 99, 96, 90, 87, and 62 g were inserted in a given area of soil, and their impedances were measured using the designed EIT sensor system. A difference was made from the obtained homogeneous and inhomogeneous results and after normalizing those were fitted in the obtained models. The biomass results of the samples were predicted, and the calculated accuracy of the obtained models was found to be 93% or above. The models have frequency dependency, and those were satisfied by the measurements at different frequencies.

## 3. Discussion

Electrical impedance imaging is a highly nonlinear and ill-posed inverse problem in which a minimization algorithm is used to obtain its approximate solution [15,16]. The common EIT inverse algorithms for finite element method (FEM) modeling are Gauss–Newton (GN), Shefield back-projection (BP), and total variation (TV), respectively [15,16,29]. GN is an iterative algorithm to solve nonlinear least square problems, being computationally inexpensive and supporting a high frame rate [15]. One-step GN is a direct linear reconstruction method commonly used for real-time imaging with a very short computation time and is one of the most widely used reconstruction approaches [16]. BP is a linear reconstruction algorithm which is capable of producing images of changes in conductivity, but the method is known to blur image contrasts [29]. TV is a regularization-based algorithm which allows image reconstruction using edge preservation, but the method is more complex to implement [16]. Several machine learning algorithms such as the artificial neural network (ANN), least angle regression (LARS), and elastic net were investigated previously to solve EIT inverse problem in real-time [30,31,32]. A long training time and relatively long reconstruction time are required when using ANN. On the other hand, LARS and elastic net seem to be less accurate, especially for real measurement data, but they are much faster than ANN [30]. A good reconstruction was made using the modified ANN compared to the modified LARS and elastic net. All the algorithms were found to be suitable for 2D imaging, but most of them have some limitations in obtaining 3D images. GN: NOSER was found to be faster in computation and suitable for 3D image reconstruction with less noise and high accuracy.

**Figure 7:**
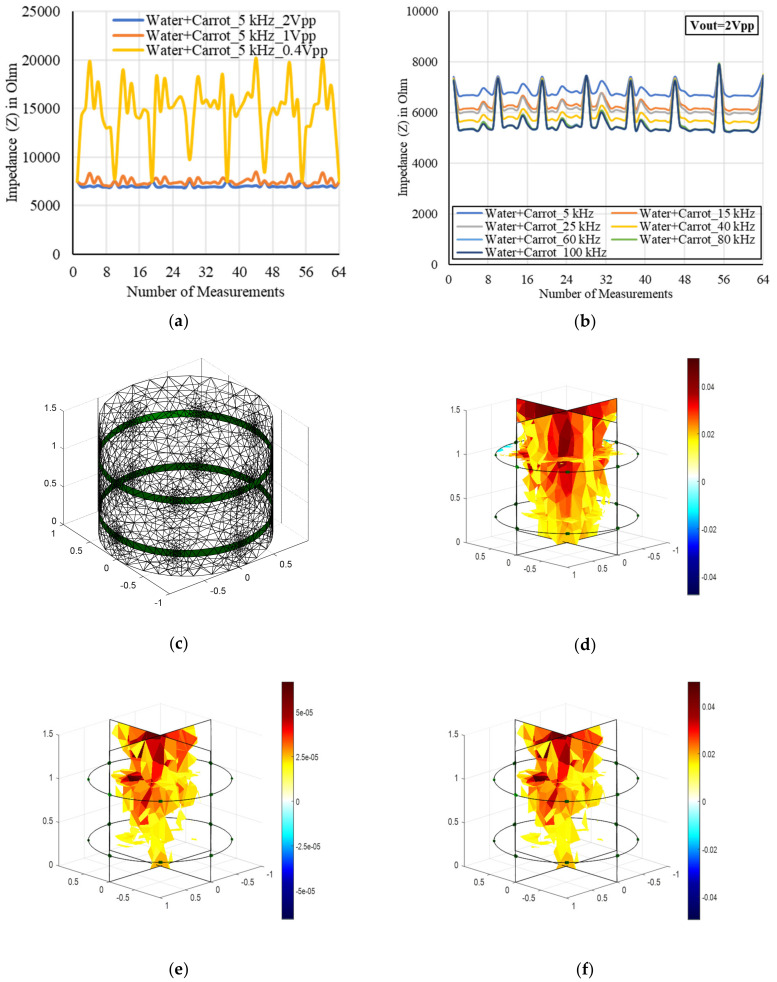
Impedance spectroscopy for an inhomogeneous media (water + carrot) in the domain by varying (**a**) output excitation (0.4–2 Vpp), and (**b**) frequency (5–100 kHz) obtained from bottom layer of the electrode array. (**c**) Netgen 3D FEM mesh for two layers of electrode array. A tomography of 6.5 inch carrot at 5 kHz, 1 mA, and 2 Vpp excitation considering (**d**) GN: NOSER (*λ* = 2.17), (**e**) Tikhonov (*λ* = 2.17), and (**f**) Tikhonov (*λ* = 0.078). (**d**–**f**) The scale represents the changes in conductivity. The reconstructed result was obtained by measuring the impedances from middle and bottom layers of the electrode array.

**Figure 8:**
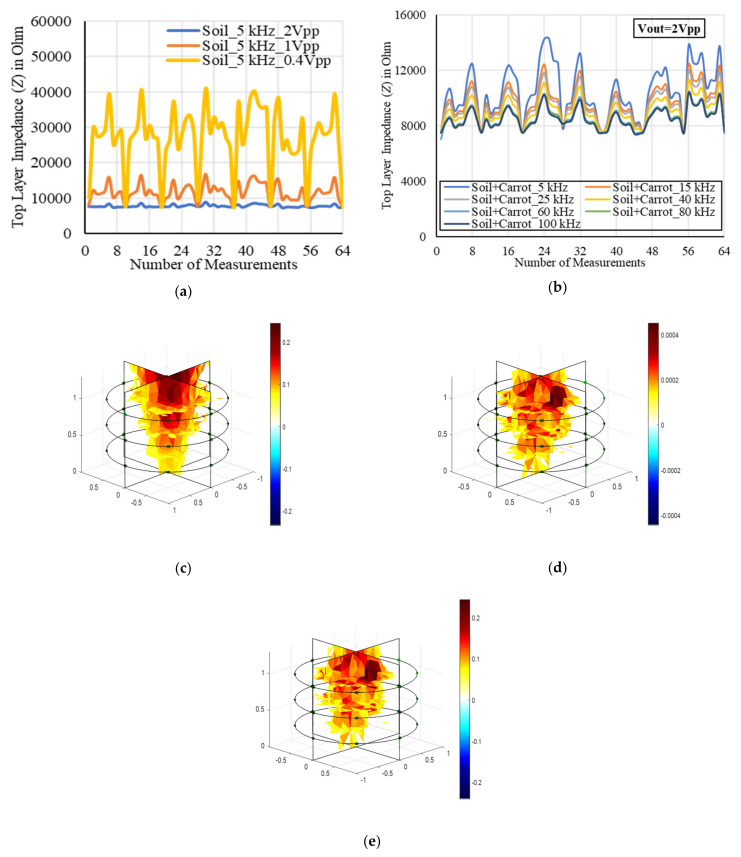
(**a**) Measured impedances from the top layer of soil electrodes by varying output excitation at 5 kHz. Impedance was decreased by increasing the output excitation and a more stable output impedance was found at 2 Vpp excitation. (**b**) Top layer impedances for an inhomogeneous media (soil + carrot) by varying frequencies (5 to 100 kHz) at 2 Vpp excitation. The impedance was decreased by increasing frequency. A tomography of 6.75 inch carrot at 5 kHz, 1 mA, and 2 Vpp excitation considering (**c**) GN: NOSER (*λ* = 2.17), (**d**) Tikhonov (*λ* = 2.17), and (**e**) Tikhonov (*λ* = 0.09). (**c**–**e**) The scale represents the changes in conductivity. A good reconstruction of the carrot was made by measuring the impedances from top, middle, and bottom layers of the electrode array.

The EIS characteristics for a root inhomogeneity in water/soil media and the corresponding tomography results were examined as shown in Figure 7 and Figure 8, respectively. The reconstruction results of the inhomogeneity were found to be satisfactory using the one-step Gauss–Newton algorithm. Three-dimensional modeling was possible using NOSER and Tikhonov regularizations with an optimized hyperparameter in addition with appropriate stimulations. The overfitting in data can be removed to obtain the smooth characteristics using the regularization techniques. A good tomography for a 6.75 inch carrot root in soil was found using NOSER (λ = 2.17) for 5 kHz and 1 mA as shown in Figure 8c.

The growth and development of a root system was evaluated by measuring impedances using the EIT sensor. Layer-wise changes in conductivity were obtained for a given carrot root as shown in Figure 2. The shape of different sized root samples and the corresponding variation in conductivity were obtained by the tomography results. The increase in changes in conductivity with the increase of dimensions of the root indicated the possible growth of the root. Impedance was found to be a good indicator in estimating the root biomass in different conditions with a good correlation as shown in Figure 1, Figure 3, Figure 4, and Figure 5, respectively. The predicted models were found to be highly correlated with *R*^2^ = 0.98 or above with lower RMSE (less than 4) at 5–100 kHz as shown in Table 1 and the models were validated with 7% or less absolute error as shown in Table 2.

In the soil–root–electrode continuum, the impedance may vary by the variation of root distribution in soil, root density, and the electrodes position from the root. The conductivity of soil particles depends on soil texture and structure, compaction, and moisture content. A difference in impedance measurements was observed between wet and dry soil. In dry soil conditions, the impedance spectra fluctuate. The test was then made using wet or high-salinity soil conditions and a solution was reached by obtaining the similar shape of the EIS spectra with a uniform impedance distribution. To enable the EIT current to pass between electrodes, the moisture was controlled in the soil media by irrigating the container before the measurements. A set of measurements was taken in an hour and the measurement accuracy was evaluated. A proper and careful irrigation is required to obtain the satisfactory results, and all the measurements were taken in similar way.

The electrode polarization may occur at the soil–electrode interface which may be resistant to flow the current freely depending on frequency [24]. The uses of insulated electrodes may reduce the polarization effects. The measurement using the EIT sensor system is found to be reliable, flexible, and repeatable. In a measurement, multiple EIS readings were taken for a time duration and the average was considered with less error. The measurement method using an EIS tool (AD5933) connected to the sensor was found to be rapid, and less time was required for computation. The proposed models were found to be robust and applicable for a wide range of frequencies. EIT is found to be very sensitive to the physiological process of the root. The multifrequency EIT performed well compared to resistance or capacitance tomography for achieving a detail information of the root [10,11,12]. In addition, more useful information of the root can be obtained considering time difference or frequency difference EIT [7,22].

**Figure 9:**
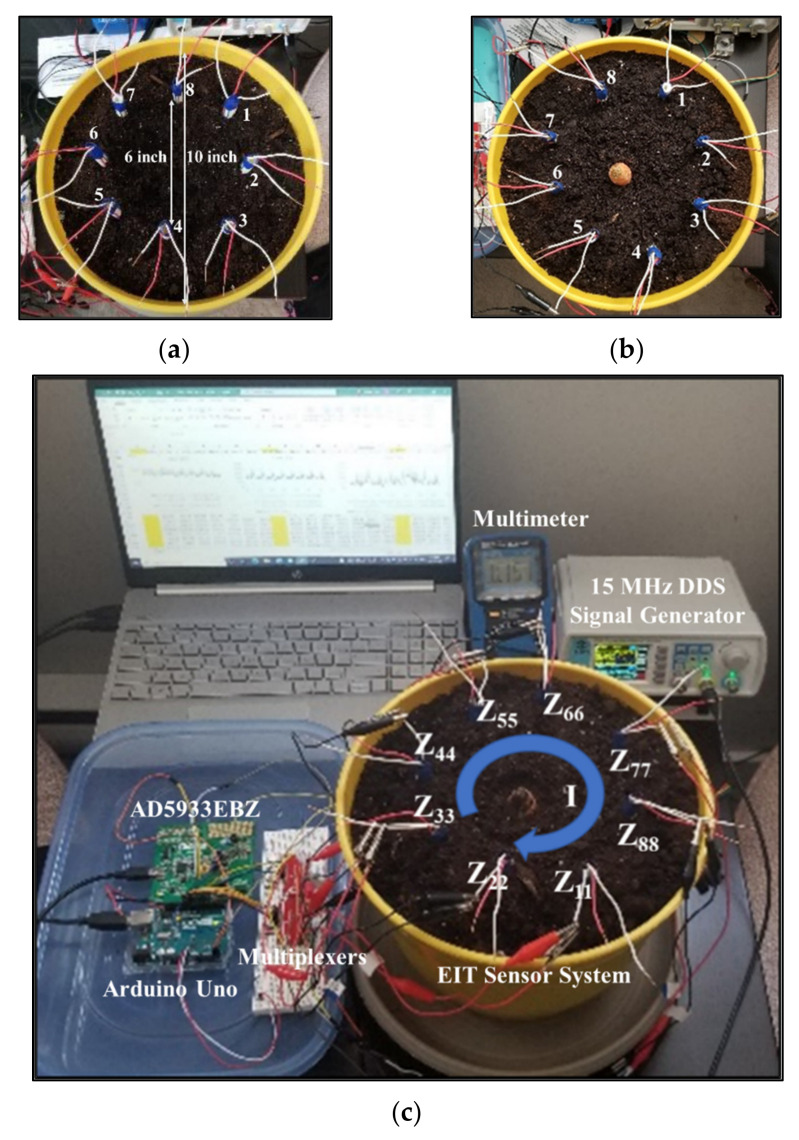
(**a**) Homogeneous media, (**b**) inhomogeneous media, and (**c**) 3D EIT experimental setup for biomass estimation of tap roots in soil. Z_11_–Z_88_ are the self-impedances measured from the electrode ports 1–1 to 8–8, and *I* is the current injected through the arrangement of circular electrodes. (**a**) Homogeneous (Soil); (**b**) Inhomogeneous (Soil + Carrot); (**c**) EIT measurement setup for carrot root biomass.

An additional investigation into the evaluation of tap root growth was made considering time-difference EIT (tdEIT) in line with the results obtained in Figure 1 and Figure 2. Another tap root of potato plant species was grown from tuber in soil at room temperature (20 °C) in a controlled environment. Day-wise the impedances were measured using the EIS tool connected to the designed EIT sensor by varying frequencies (5–100 kHz) considering an experimental setup similar to Figure 9c. The variation in root impedance and the corresponding conductivity was found during growth depending on root length and weight. Initially, the root conductivity was low when it was expanded horizontally, and in vegetative stage the conductivity was improved by increasing the surface area and weight when the root was expanded vertically. The observation was found to be similar to the results concluded in Figure 1 and Figure 2. In addition, a good observation was made from the results obtained using tdEIT. Significant changes in calculated conductivity over time were observed and found to increase with time. The results indicated the growth of the potato tap root and the method was validated. Also, the method for biomass estimation can be extended to multiple potato roots or the roots of other plant species and further investigation can be made in obtaining the models.

The developed EIT system is found to be less expensive, and suitable for in situ measurements in both water and soil media, which shows an advancement of the previous works [10,11,12]. In addition, the designed sensor is capable for obtaining 3D information of the roots using a lower sized electrode array. In another work, Postic et al. proposed a rapid estimation of root biomass for wheat plants by measuring capacity using a handheld LCR meter (up to 20 kHz) [26]. The method was required an expensive instrumentation and obtained $R^{2}<90\%$ for soil media. A comparative study was made with other EIT sensor systems for root analysis and shown in Table 3.

## 4. Materials and Methods

### 4.1. Design of EIT Sensor System

**Figure 10:**
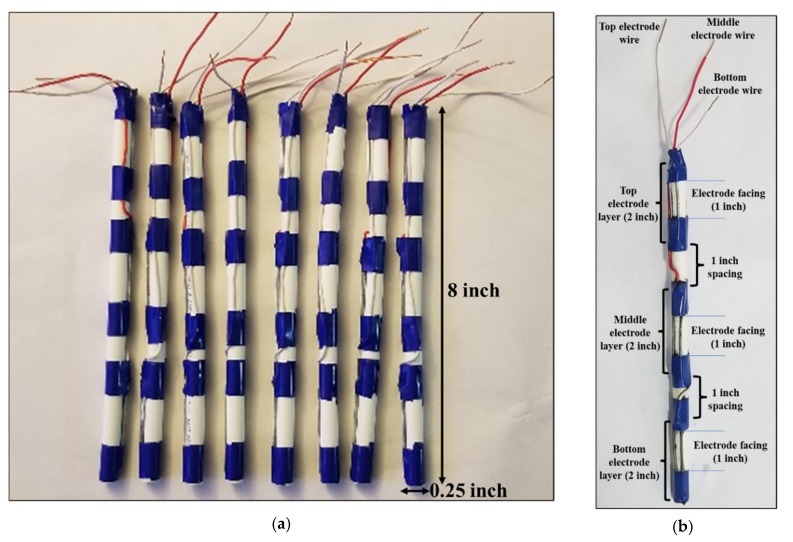
(**a**) Electrode array for designing an in situ 3D EIT system using 24 electrodes in eight plastic sticks of three layers each, and (**b**) distributed electrodes in three layers (top, middle, and bottom) of a plastic stick.

A new EIT sensor system was designed using an electrode array considering three-layer of electrodes for in situ measurements. Eight plastic sticks of 8 inches in length and 0.25 inches in diameter were taken. A total of 24 electrodes with 3 electrodes per stick were configured in three different layers of top, middle, and bottom positions as shown in Figure 10. The electrodes were numbered sequentially in the three layers of array and the measurements were carried out in the 3D domain. The type and size of the electrodes were optimized by several experiments considering previous research works [15,33,34]. Steel electrodes were found to be suitable with good current carrying capability and a good correlation was found between the measured impedances and dimensions (length, diameter) of the electrodes. Steel material is comparatively less expensive than highly conductive materials such as silver, copper, or aluminum. In this work, a low-cost sensor was designed by using the steel electrodes in the array.

The selected metal electrode length was 2 inches with a diameter of 0.0625 inches each. The spacing between the two adjacent layers of electrodes in the stick was considered 1 inch. Three layers of electrodes were connected to three different wires to establish the probe connection for the measurements. A good conductivity distribution was found with the optimized dimensions of the electrodes and the electrode array system was found to be suitable for utilization in both water and soil media considering a planar-aligned electrode placement configuration [34]. The designed electrode array is dynamic and adjustable which increased the suitability for the in situ measurements. In different experiments of this work, the spacing between two adjacent sticks was optimized as 2.5 inches for placing in a circle of the water or soil media.

### 4.2. Development of EIT Data Acquisition System

**Figure 11:**
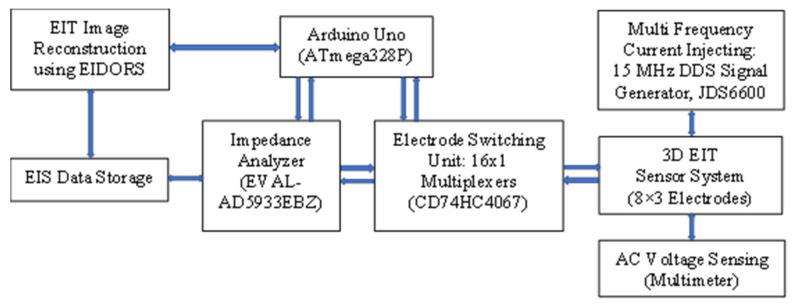
A developed EIT data acquisition system using the designed electrode array for 3D imaging.

An automated EIT data acquisition system is developed for 3D operations as shown in Figure 11. An EIS tool (EVAL-AD5933EBZ) was interfaced with the designed EIT electrode array system, in addition with two electrode switching multiplexers (CD74HC4067), Arduino Uno (ATmega328P), EIS data storage (PuTTY), and PC, respectively. AD5933 with on-board frequency generator is a high precision impedance converter system, and it has a programmable graphic user interface with frequency sweep capability and serial I^2^C interface [35,36]. At first, the current carrying capability of the electrodes was checked by injecting current through the driving electrodes at different frequencies using a 15 MHz DDS signal generator (JDS6600). A four-pole method was applied, and the ac sensing voltages were measured by the sensing electrodes using a multimeter. The current and voltage levels in multiple electrodes of the array were examined by varying stimulation methods. Once the functionality of the sensor was tested it was utilized for the measurements in different experiments.

The impedance in the EIT domain was measured by the current injected through the multiple electrodes with an automated and appropriate EIS measurement setting using Arduino Uno programming. A two-pole method was applied and the measured impedances by the electrodes in the array were stored in the data storage for different frequencies of 1 kHz to 100 kHz. The measurements were made using the output excitations of 0.2 to 2 Vpp controlled by the EIS tool (AD5933). The measured impedances of the electrodes were found to be very sensitive to the two-pole measurements for any given object in the EIT domain. Finally, the data stored using the acquisition system was analyzed for EIT image reconstruction using EIDORS [3,13,15].

### 4.3. Modeling and Calculating Conductivity

**Figure 12:**
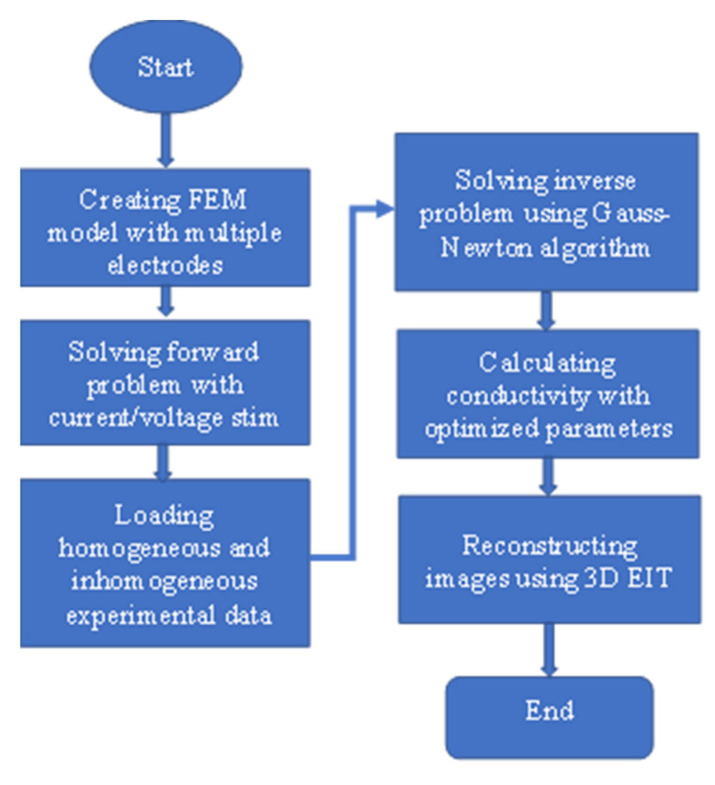
Flowchart of EIDORS operation with the target of image reconstruction by calculating conductivity using 3D EIT.

**Figure 13:**
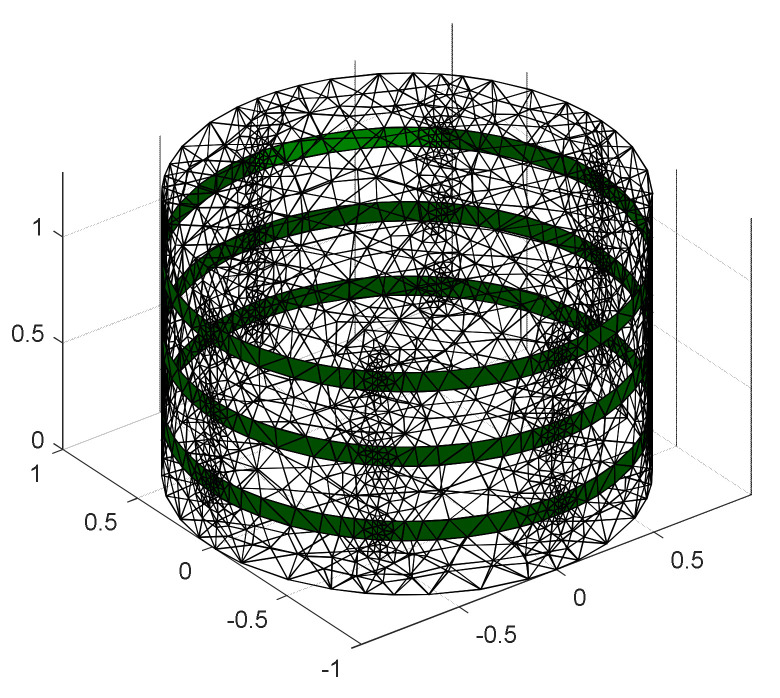
Netgen 3D FEM mesh for three layers of electrode array—a cylindrical domain to reconstruct the image using EIDORS in MATLAB.

EIT problems were solved numerically considering a finite element method (FEM) modeling using an open source software, EIDORS in MATLAB. The flowchart of EIDORS operation is presented in Figure 12. In addition, a Netgen cylindrical model of ‘ng_mk_cyl_models’ was considered for modeling in a 3D domain. The model consisted of 4020 nodes, 17,284 elements, and 4034 boundaries considering 24 electrodes distributed in three layers of the array (8 electrodes per layer). The Netgen FEM mesh for the reconstruction in a cylindrical domain of 3D EIT is presented in Figure 13. The spatial resolution of EIT can be improved by selecting the correct drive pattern in the model. The current/voltage stimulation was made by selecting the electrode ports appropriately in the array. The model was tested with different drive patterns and the reconstruction results were evaluated.

A complete electrode model was used to relate the internal conductivity distribution to the boundary voltage measurement by the injected current through the electrodes of an EIT system. The conductivity distribution σ is known by the electric potential *V* in the EIT domain Ω for *L* number of electrodes. The EIT governing equation can be described by Laplace’s equation (derived from Maxwell’s equation) as [2,9]

(10) $$\nabla \cdot \left(\sigma \nabla V\right)=0\;\;\mathrm{in}\mathrm{domain}\;\Omega$$

The conductivity map of an object in a single step can be calculated by knowing the boundary voltage difference for the given injected current as [2,9,20]

(11) $$\Delta \sigma ={\left(J^{T}WJ+\lambda^{2}R\right)}^{-1}J^{T}W\Delta V$$

where *λ* is the hyperparameter that controls the trade-off between resolution and noise attenuation, *W* is the inverse of the covariance of measurements, *R* is an estimation of the inverse of the noise covariance, and *J* is the Jacobian which is a determinant for the measurement of voltage sensitivity. The calculation of the Jacobian matrix depends on the current injection in a 3D electrode array system. The optimum conductivity change is calculated by the voltage difference, $\Delta V=V_{i}-V_{h}$, where *V_h_* and *V_i_* are the boundary voltages for homogeneous and inhomogeneous media.

The changes in conductivity were obtained by the following steps: (i) model selection, (ii) stimulation, (iii) loading experimental data, and (iv) calculating the conductivity using one-step Gauss–Newton (GN) algorithm such as prior NOSER (Newton’s one-step error reconstructor). In difference imaging, the Gauss–Newton method can be used to minimize the differences between homogeneous and inhomogeneous data. The GN method was employed as an inverse solver to reconstruct the internal conductivity distribution for 3D modeling. GN: NOSER performed well, and good observations were made from the tomography results compared to the other regularization methods such as Tikhonov, Laplacian, and Gaussian high-pass filtering (GHPF), respectively. A satisfactory result was also found using Tikhonov but with a limited capacity. On the other hand, no satisfactory result was found using Laplacian, and GHPF was not able to generate 3D models.

In the forward solution, the experimental data obtained from homogeneous and inhomogeneous measurements for the given 3D EIT domain were utilized in the cylindrical FEM mesh using EIDORS with an appropriate stimulation, and a difference method of reconstruction was applied using an optimized model in the inverse solution. The size of the domain, inhomogeneity position and size, stimulation current/voltage (*I*/*V*), frequency (*f*), and noise controlled hyperparameter (*λ*) values were optimized for calculating the changes in conductivity and reconstructing the image of the given inhomogeneity in the domain.

### 4.4. Sensor Characterization

After designing, the EIT sensor was tested and characterized by the injected current. The conductivity distribution was evaluated for multiple inhomogeneities in the domain. The current distribution through the electrodes was checked for different voltage levels at multiple frequencies. Different sensing methods were applied, and the dimensional dependency on the measurements of the inhomogeneity was evaluated. Later, the stimulation current was fixed at 1 mA and the boundary voltages were calculated by measuring impedances in homogeneous and inhomogeneous media at different frequencies, and the difference of those was used to map the conductivity of the inhomogeneity in the domain.

The EIS measurements of the samples were carried out at room temperature (20 °C) in a controlled environment using the designed EIT sensor system and the reconstruction analysis was performed. Carrot (*Daucus carota* L.) root was used in the measurements as a sample of tap root system. Carrot is a widely cultivated edible plant species with a variety of shapes, sizes, and colors. The carrot roots have good storage ability and contain abundant biologically active substances. The carrot root is reach in minerals and antioxidants and is a good source of carotenoids (natural pigments of photosynthetic organisms). Carrots are becoming more popular due to their abundant nutrients and benefits for medical applications. In addition, carrot root has a good conductivity distribution in a wide range of frequencies and has been found to be suitable for the study of the growth and development in plant biology. Hence, the carrots were chosen as a biological material instead of other root plants. The electrical impedance (*Z*) of a sample measured by the sensor connected to EIS tool (AD5933) is related to the DFT magnitude of $\sqrt{R^{2}+X^{2}}$ and gain factor as follows [35,36]:

(12) $$Impedance,\;Z\left(Ohm\right)=\frac{1}{Gain\;Factor\times \sqrt{R^{2}+X^{2}}}$$

where the gain factor is calibrated by a known resistance of 7.5 kΩ. The gain factor varies with the variation of output voltage excitation (*V_out_*) and physical frequency (*f*) for a given sample. Here, *R* and *X* are the DFT real and imaginary outputs registered at different frequency codes generated by the physical frequency, $f=f_{clk}\times Frequency\;Code/2^{29}$, where *f_clk_* is the master clock frequency of 16.776 MHz for the internal oscillation [35].

Initially, the functionality of AD5933 was examined in a controlled environment at room temperature by employing the two-electrode method. A pair of electrocardiogram (ECG) electrodes connected to the EIS tool were separated by *d* distance and the capacitive reactance, *X_c_* of a sample was calculated as $X_{c}=1/2\pi fC$, the sample capacitance, C=εA/d, where *A* is the cross-sectional area, and *ε* is the medium constant. The reactance is related to the impedance of the sample as $X_{c}=Z\sin\theta$, where *θ* is the phase of the electrical impedance. In this work, the magnitude of impedance obtained from AD5933 was taken for analysis in different experiments. The influence of the impedance magnitude in determining the sample characteristics and modeling was found to be significantly higher than the phase. The impedance of a sample is dependent on frequency, and the spectroscopy used to determine the sample characteristics can be obtained for a wide range of frequencies. The EIS characteristics of the sample were obtained by varying frequencies up to 100 kHz using the EIS tool.

**Figure 14:**
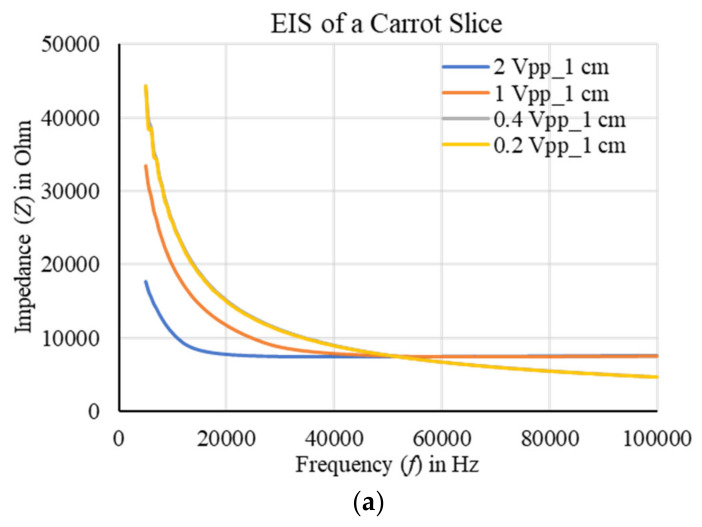

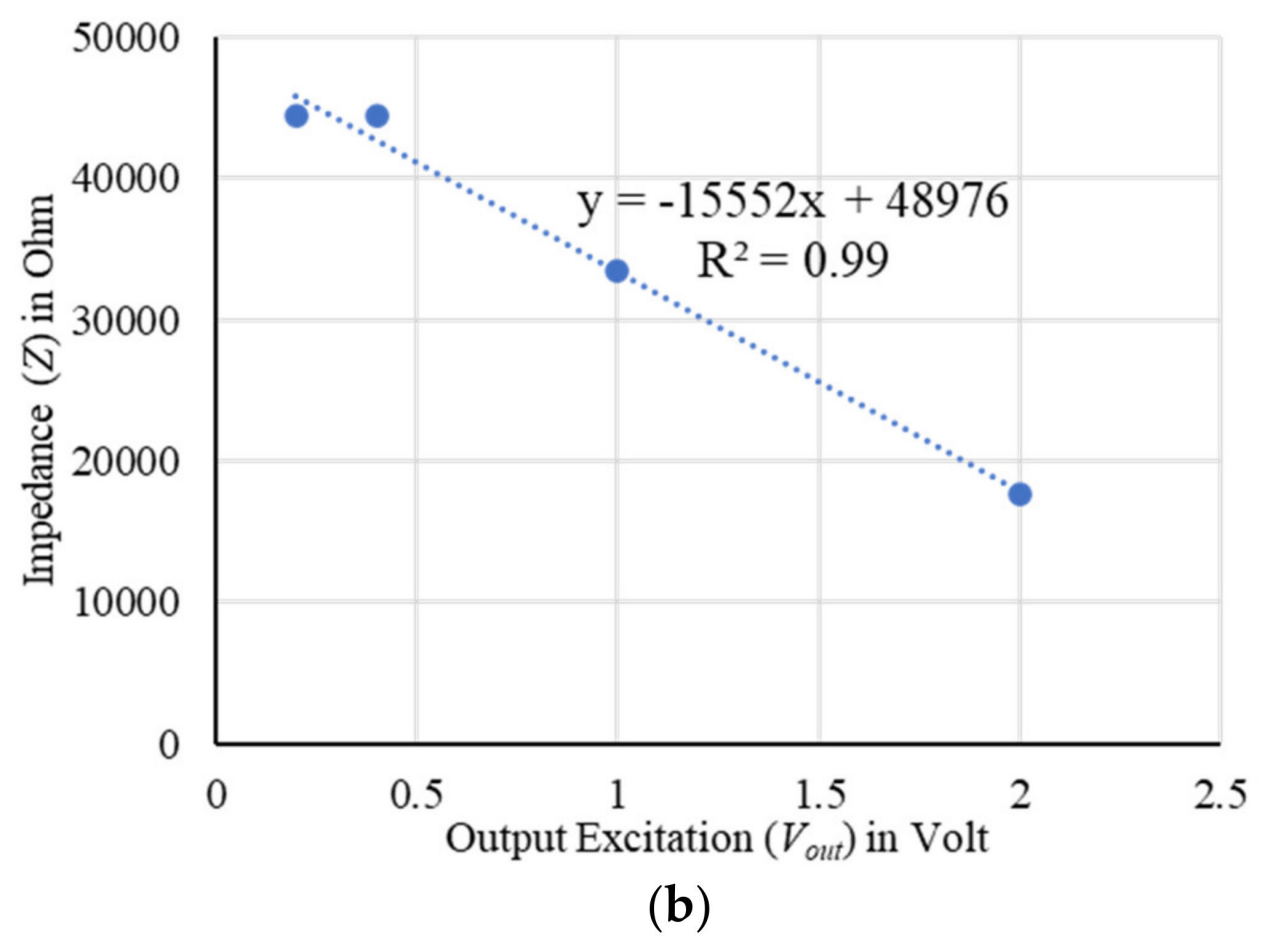
(**a**) EIS of a carrot slice using the two-electrode method by varying output excitation, and (**b**) the corresponding correlation at 5 kHz. No variation was observed below 0.4 Vpp excitation because of saturated output. A good correlation with a stable output was found for 2 Vpp excitation considering 1 cm spacing of the electrodes.

**Figure 15:**
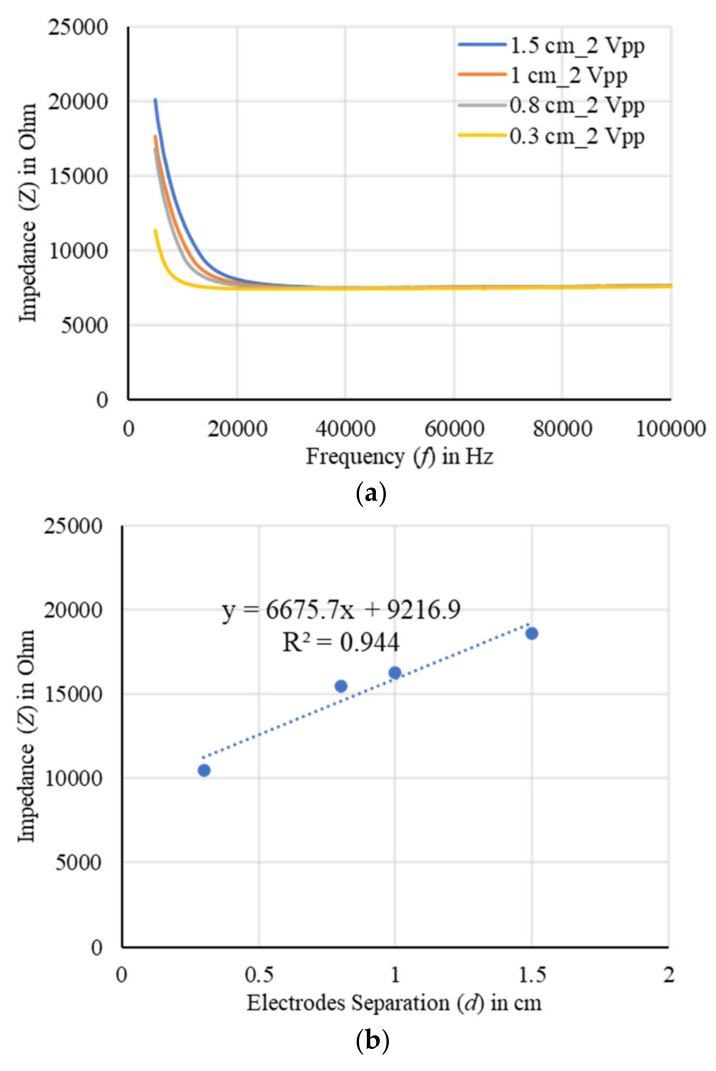
(**a**) EIS of a carrot slice using the two-electrode method by varying separation of the electrodes, and (**b**) the corresponding correlation at 5 kHz. A good correlation was found for 1 cm spacing of the electrodes considering 2 Vpp excitation.

The impedances for a carrot slice of 0.2 inches in length and 1 inch in diameter were measured by varying the output excitation from 0.2 to 2 Vpp and spacing between two electrodes from 0.3 to 1.5 cm. A good correlation with a stable output was found for 2 Vpp excitation and 1 cm spacing of the electrodes as shown in Figure 14 and Figure 15, respectively. More than 97% correlation was found, and the impedance profile indicated a good conductivity distribution with the optimized output excitation and spacing of the electrodes. The sample impedance was decreased with the increase of output excitation, on the other hand, the impedance was increased with the increase of electrodes separation. In addition, the sample was found to be more conductive at the high frequencies. It was observed that the impedance was not significantly varied at 0.2 Vpp excitation, hence, in further experiments the output excitation was not taken below 0.4 Vpp.

**Figure 16:**
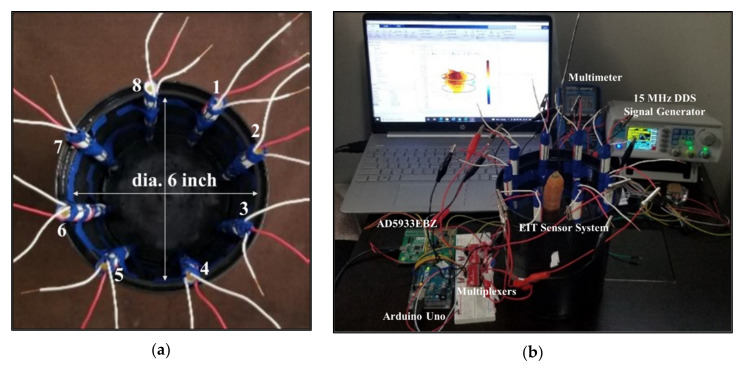
(**a**) EIT electrode array sensor system, and (**b**) experimental setup for EIT sensor characterization using electrodes in water media and a carrot of 6.5 inches in length. During the measurements the carrot was fully immersed in water. The visibility of the sample may vary based on the length of the sample with respect to domain height.

Next, the designed EIT sensor system was characterized using a tap root system such as carrot root in water media. The electrode sticks were placed in a black cylindrical plastic domain of 7 inches in height and 6 inches in diameter as shown in Figure 16. The adjacent spacing between the sticks was 2.5 inches. The homogeneous media was created by filling the domain with water. The top layer could not be fully immersed in water because of the greater height of the stick, but the middle and bottom layers of electrodes were fully immersed. A planar-aligned electrode placement configuration was chosen for 3D EIT imaging where the electrodes were placed vertically in the same line of the domain and the measurement method with this arrangement was found to be robust in obtaining tomography with less noise. The electrodes were configured and numbered sequentially in different layers, and the measurements were taken with the target of obtaining tomography of a carrot of 6.5 inches in length. The sensor system with AD5933 was characterized by varying the excitation from 0.4 to 2 Vpp at different frequencies. The impedances were measured with and without the carrot, and a difference was applied to obtain the conductivity of the carrot. Although a partial immersion of the carrot is shown in Figure 9b to make the sample visible, the carrot was fully immersed in water when the inhomogeneous measurements were taken.

The EIS measurements were taken at different frequencies of 5 kHz to 100 kHz. A total of 64 measurements (1–1, 1–2, 1–3, 1–4, 1–5, 1–6, 1–7, and 1–8 with respect to electrode 1; 2–1, 2–2, 2–3, 2–4, 2–5, 2–6, 2–7, and 2–8 with respect to electrode 2, and so on) were taken from eight electrodes in one layer. Similarly, the measurements were taken in other layers of the array. The variation of impedances was found to be sinusoid at different electrode positions as shown in Figure 7. The output excitation was varied at different frequencies for the measurements in the top to bottom layers of the EIT electrode array. The impedance was decreased with the increase of frequency. The measured impedance was high at a low excitation of 0.4 Vpp when the frequency was fixed, and an oscillated output was observed due to the oscillation of gain and DFT outputs at a given stimulation current. A high oscillation was found at low frequency of 5 kHz, and the effect was reduced at the high frequencies. On the other hand, the output with lower impedances was found to be stable at a higher excitation of 2 Vpp for low or high frequencies and all the measurements in the further experiments of this work were carried out accordingly.

A trade-off between output excitation and gain factor was found on obtaining stable output for the given frequency and operating voltage of 2.7–5.5 V of AD5933. The impedance was decreased by increasing the output excitation and frequency as shown in Figure 7. A more stable output was found at a high excitation of 2 Vpp. Although the measurements were taken from top to bottom layers of the electrode array, the measured data obtained from middle and bottom layers of electrodes were only taken to reconstruct the image of the carrot inhomogeneity. A Netgen 3D FEM mesh for two layers of electrode array was generated using EIDORS as shown in Figure 7c. The model consisted of 3287 nodes, 14,296 elements, and 3198 boundaries considering 16 electrodes distributed in two layers of the array (8 electrodes per layer). A tomography of the carrot was obtained at 5 kHz, and 1 mA considering GN: NOSER (*λ* = 2.17) in the inverse model as shown in Figure 7d. A total of 208 boundary voltages obtained from the individual measurement of homogeneous and inhomogeneous media were utilized to obtain the reconstructed conductivity image of the sample. A conductive behavior of the carrot was found for the given stimulation current and frequency. After normalizing the boundary voltages, the maximum change in conductivity of 0.05 was calculated. The changes in conductivity for the given sample were varied using Tikhonov and optimized by varying hyperparameter value. Overall, 3D information of the sample was obtained showing the variation in conductivity level at different positions according to the shape and size of the sample. Similarly, the observation can be made in other frequencies and the spectral variations can be studied. To overcome the limitations in obtaining good tomography a new plastic domain of longer height can be chosen in further experiments.

Later, a new plastic container of 8 inches in height and 10 inches in diameter was taken for the sensor characterization in soil media with the target of obtaining better reconstructed results by improving the accuracy in the shape and size of the root samples and to fulfill the main objective of the work. The container was filled with 10.8 L soil (contains a combination of humus, peat moss, sand, and perlite) and the designed EIT electrode array was placed in the soil in a circle of 6 inches in diameter as shown in Figure 9. In the soil–electrode continuum, the measurements in the soil are dependent on the position and distance of the electrodes from each other, and insertion depth into the soil. The spacing between two adjacent sticks was optimized as 2.5 inches and the electrodes in three different layers were configured for the measurements sequentially. A planar-aligned electrode configuration was made for 3D imaging. In order to obtain the uniform distribution of impedances, the electrodes facings of the sticks in the array were maintained with appropriate alignment.

The EIT sensor system was tested and characterized by the injected current in soil media. Different sensing methods were applied, and the distributed conductivity in the 3D EIT domain was calculated by the obtained boundary voltages in multiple frequencies. The voltages were obtained from the measured electrical impedances considering 1 mA current at the boundary. During the characterization, it was found that the impedance pattern may vary (i) if the electrode position changes horizontally, (ii) if the electrode position changes vertically, and (iii) if the diameter of the circle in soil media of the domain changes. Layer-wise from top to bottom of the electrode array, the EIS measurements were taken for different frequencies of 5 kHz to 100 kHz by varying the output excitation from 0.4 Vpp to 2 Vpp. The measurements were taken for homogeneous (soil) and inhomogeneous (soil + carrot) media using a new carrot sample of 6.75 inches in length, and the results obtained from the top layer of the electrode array are presented in Figure 8. The impedance was decreased by increasing the excitation and frequency. The result was found to be more stable at the high excitation of 2 Vpp.

Soil moisture is one of the most important factors which may affect the measured impedances and that was taken in consideration. Electrical impedance measurements are very sensitive to the soil moisture content [27]. Hence, a difference was made between inhomogeneous and homogeneous measurements to obtain the actual impedance of the carrot. A tomography result of the carrot sample was obtained by measuring impedances from top to bottom layers of the electrode array and obtaining the normalized voltage difference. A total of 504 boundary voltages obtained from the individual measurement of homogeneous and inhomogeneous media were utilized to obtain the reconstructed conductivity image of the sample. The maximum changes in conductivity of 0.24 was obtained at 5 kHz, and 1 mA considering GN: NOSER (*λ* = 2.17) in the inverse model as shown in Figure 8c. An optimized conductivity result was obtained using Tikhonov also by varying the hyperparameter value. In overall, a good tomography was found with better shape and size of the sample considering 1 mA stimulation current for three layers of electrode array. EIDORS operation was made to obtain the reconstructed results, and similar considerations for modeling, framing, and scaling using Netgen FEM mesh were made for further experiments in this work. The confinement in the EIT domain was improved with higher numbers of nodes, elements, and boundaries considering 24 electrodes distributed in three layers of the array (8 electrodes per layer) and the error in size obtained by the reconstructed image against the real carrot size was reduced. The error in size of the sample can be further minimized by increasing the number of layers of electrodes in the sensor array.

### 4.5. Data Process and Analysis

The biomass estimation of tap roots was made at room temperature (20 °C) in a controlled environment. Multiple carrot roots were taken in different experiments as the samples of tap roots and their EIS measurements were carried out. The EIS data taken in multiple ports of the EIT electrode array sensor were stored using an open source software PuTTY (interfaced with Arduino Uno COM3 port). A statistical analysis was performed with the obtained data using PrimaXL Data Analysis ToolPak [36]. A multiple linear regression analysis was carried out considering the least square method (the most common method of estimation in machine learning) using Equation (13).

(13) $$\hat{Y}=\omega_{0}+\omega_{1}Z_{f1}+\omega_{2}Z_{f2}+\ldots +\omega_{k}Z_{fk}$$

where *Z_f_*_1_, *Z_f_*_2_, …, *Z_fk_* are the measured average impedances for *k* number of features of *f*1 to *fk*. The intercept is $\omega_{0},$ and $\omega_{1},\;\omega_{2},\ldots ,\;\omega_{k}$ are the coefficients.

Seven features of 5, 15, 25, 40, 60, 80, and 100 kHz were taken in the dataset. First, the multicollinearity problem was examined. A dataset suffers from multicollinearity: (i) if the correlation coefficient (*R*) between the explanatory variables is close to 1, (ii) if there is no change in coefficient of determination (*R*^2^) after adding an independent variable, and (iii) if the tolerance value (TV = 1 − *R*^2^) is less than 0.1 and the variance inflation factor (VIF = 1/TV) is greater than 10. The highly correlated features of more than 95% correlation coefficient were removed. Later, the Wrapper backward elimination method was applied considering the probability of rejection of null hypothesis *p* ≤ 0.05 using an individual T-test [36]. After several iterations, the training and validation was performed considering the overall F-test (*p* ≤ 0.05) and the features were selected accordingly.

In addition, layer-wise mean (m) and standard deviation (SD) of the measured impedances were taken and the models were evaluated for different frequencies. The obtained data suffered from multicollinearity and ridge regression was applied with the help of Equation (14).

(14) $$\hat{Y}=\omega_{0}+\omega_{1}Z_{l1m}+\omega_{2}Z_{l1SD}+\omega_{3}Z_{l2m}+\omega_{4}Z_{l2SD}+\omega_{5}Z_{l3m}+\omega_{6}Z_{l3SD}$$

where the intercept is $\omega_{0},$ and $\omega_{1},\omega_{2},\omega_{3},\omega_{4},\;\omega_{5},\;\omega_{6}$ are the coefficients. *Z*_l1*m*_, *Z*_l2*m*_, *Z*_l3*m*_ are the mean values, and *Z*_l1*SD*_, *Z*_l2*SD*_, *Z*_l3*SD*_ are the standard deviation of impedances in layer one, two, and three, respectively.

Ridge regression addresses some of the problems of ordinary least squares by imposing a penalty on the size of coefficients with the target of minimizing the cost function. The ridge coefficients minimize a penalized residual sum of squares, $\underset{\omega }{\min}\|y-Z\omega {\|}_{2}^{2}+\alpha \|\omega {\|}_{2}^{2}$. Here, α≥0 is a complexity parameter that controls the amount of shrinkage: the larger the value of α, the greater the amount of shrinkage and thus the coefficients become more robust to collinearity. The ridge includes all the predictors in the final model which are more relevant and useful. The method utilized the regularization technique to prevent the multicollinearity. The model complexity was reduced by reducing the variance of the model significantly.

## 5. Conclusions

A novel, dynamic, and adjustable EIT electrode array sensor system is designed for developing a rapid, cost-effective, and radiation-free multifrequency 3D EIT data acquisition system. The EIT system can be applied in both controlled settings and in the field. A non-destructive evaluation of biomass estimation of tap roots is carried out by measuring impedances using the designed EIT sensor system. The root system is characterized and monitored by the sensor in a controlled environment at room temperature, and non-invasive 3D imaging is performed in both water and soil media. The EIT sensor is found to be capable of evaluating the growth of the root by calculating the changes in conductivity and obtaining the associated information from tomography. A strong correlation is found between the biomass and measured impedance of the root and several models are developed for biomass estimation of the carrot roots. The obtained models are validated with less error, and the tomographic images of the root systems are generated in high spectral ranges to obtain more useful information on the growth and development of the roots. The proposed EIT sensor system can be explored further in a field setting for estimating root biomass in different growth stages.

## Figures and Tables

**Table 1:** Regression performance for predicting carrot root biomass in different frequencies (probability of rejection, *p* ≤ 0.05).

Features	*R* ^2^	Adj. *R*^2^	RMSE	*p*-Value
5 kHz	0.994	0.976	2.58	0.017
15 kHz	0.986	0.945	3.94	0.04
25 kHz	0.987	0.949	3.81	0.037
40 kHz	0.99	0.963	3.26	0.027
60 kHz	0.988	0.951	3.72	0.035
80 kHz	0.988	0.954	3.61	0.033
100 kHz	0.989	0.959	3.43	0.03

**Table 2:** Validation of EIT regression models for predicting carrot actual biomass weight in a given area of soil.

New Carrot Samples	Actual Biomass Weight (g)	Predicted Biomass Weight (g)	Absolute Error (%)
Sample 1	142	132.034	7.01
Sample 2	115	119.217	3.66
Sample 3	99	102.587	3.62
Sample 4	96	94.67	1.38
Sample 5	90	84.17	6.47
Sample 6	87	84.9	2.41
Sample 7	62	64.68	4.32

**Table 3:** Comparison with other EIT sensor systems for root analysis.

	Weigand and Kemna [10,11]	Corona-Lopez et al. [12]	Proposed EIT Sensor (This Work)
Sensor design media	Water-filled container	Compost-filled container	Water- and soil-filled container
Type and size of electrode array	Static with 38 electrodes	Static with 32 electrodes	Dynamic and adjustable with 24 electrodes
Operating frequency (kHz)	0.00046–45	5–10	1–100
Imaging capability	Two-dimensional	Three-dimensional	Three-dimensional
Measurement sensitivity	Characterize and monitor the tap roots (oilseed)	Visualize the development of tap roots (oilseed)	Evaluate the growth and estimate the biomass of tap roots (carrot)

## Data Availability

Data is contained within the article.
